# Allicin enhances host pro-inflammatory immune responses and protects against acute murine malaria infection

**DOI:** 10.1186/1475-2875-11-268

**Published:** 2012-08-08

**Authors:** Yonghui Feng, Xiaotong Zhu, Qinghui Wang, Yongjun Jiang, Hong Shang, Liwang Cui, Yaming Cao

**Affiliations:** 1 Department of Immunology, College of Basic Medical Sciences, China Medical University, Shenyang, Liaoning, China; 2 Department of Laboratory Medicine, the First Hospital of China Medical University, Shenyang, Liaoning, China; 3 The Key Laboratory of AIDS Immunology of Ministry of Health, the First Hospital of China Medical University, Shenyang, Liaoning, China; 4 Department of Entomology, Pennsylvania State University, University Park, PA, USA

**Keywords:** Pro-inflammatory mediators, *Plasmodium yoelii*, Dendritic cells, Macrophages

## Abstract

**Background:**

During malaria infection, multiple pro-inflammatory mediators including IFN-γ, TNF and nitric oxide (NO) play a crucial role in the protection against the parasites. Modulation of host immunity is an important strategy to improve the outcome of malaria infection. Allicin is the major biologically active component of garlic and shows anti-microbial activity. Allicin is also active against protozoan parasites including *Plasmodium*, which is thought to be mediated by inhibiting cysteine proteases. In this study, the immunomodulatory activities of allicin were assessed during acute malaria infection using a rodent malaria model *Plasmodium yoelii* 17XL.

**Methods:**

To determine whether allicin modulates host immune responses against malaria infection, mice were treated with allicin after infection with *P. yoelii* 17XL. Mortality was checked daily and parasitaemia was determined every other day. Pro-inflammatory mediators and IL-4 were quantified by ELISA, while NO level was determined by the Griess method. The populations of dendritic cells (DCs), macrophages, CD4^+^ T and regulatory T cells (Treg) were assessed by FACS.

**Results:**

Allicin reduced parasitaemia and prolonged survival of the host in a dose-dependent manner. This effect is at least partially due to improved host immune responses. Results showed that allicin treatment enhanced the production of pro-inflammatory mediators such as IFN-γ, TNF, IL-12p70 and NO. The absolute numbers of CD4^+^ T cells, DCs and macrophages were significantly higher in allicin-treated mice. In addition, allicin promoted the maturation of CD11c^+^ DCs, whereas it did not cause major changes in IL-4 and the level of anti-inflammatory cytokine IL-10.

**Conclusions:**

Allicin could partially protect host against *P. yoelii* 17XL through enhancement of the host innate and adaptive immune responses.

## Background

Malaria with its ~250 million clinical cases and a human death toll of 0.9 million per year remains a huge problem in many tropical and subtropical countries [1]. To realize the ambitious goal of malaria elimination, novel integrated strategies are needed. Among them, vaccines to reduce the morbidity and mortality associated with malaria have been intensively pursued, but so far no malaria vaccine is available. Vaccine development efforts are thwarted partially by incomplete understanding of the mechanisms of protective immunity against malaria, which normally develops in populations residing in hyperendemic areas after repeated exposure to malaria infections.

To identify the key targets and mechanisms of protective immunity against malaria, experimental murine malaria models have significantly advanced our understanding of how *Plasmodium* parasites interact with the host immune responses *in vivo*[2]. It has become evident that Th1 type pro-inflammatory immune responses are essential for controlling the parasite load during the early phase of infection [3-5]. Protective CD4^+^ T cells release IFN-γ to activate effector cells such as macrophages, which may exert anti-malarial effects by releasing TNF and nitric oxide (NO) [6,7]. NO can reduce parasitaemia during the initial phase of blood-stage malaria infection [8,9]. During malaria infection, regulatory T cells (Treg) can expand and suppress the establishment of Th1 immune response [10], resulting in increased parasitaemia and mortality of the host [11,12]. Dendritic cells (DCs) are critical players in innate immunity and priming T cell-dependent, specific immune responses to malaria infection. DCs activated in the spleen are major antigen-presenting cells (APCs), and also a source of cytokines that help shape up cell-mediated and humoral immunity [13,14]. Therefore, immunomodulatory drugs that improve the functions of DCs may lead to enhanced immunity against malaria parasites.

Many natural products possess immunomodulatory activities, which have long been sought for treating human diseases. Garlic (*Allium sativum*) is one of the most ancient vegetables and its medicinal uses are dated back *>*5,000 years [15]. Garlic possesses evident pharmacological properties, such as antimicrobial [16,17], antioxidant [18,19], and anticancer activities [20,21]. Garlic and its components have potent antiparasitic activities against many human and animal parasites [22], such as *Leishmania*[23,24], *Schistosoma*[25,26], *Trypanosoma*, *Giardia*, *Entamoeba*[27,28], and *Plasmodium*[29,30]. Allicin (diallyl thiosulfinate), rapidly converted from allin by allinase in crushed fresh garlic cloves, is a major component and thiosulphinate compound responsible for the biological activity of garlic [31]. A recent study reported that the potent anti-plasmodial and anti-trypanosomal activity of allicin is associated with its inhibitory effect on the cysteine proteases of the parasites [32]. In addition to the proclaimed nutritional and antimicrobial effects [33], garlic has immunomodulatory activities [15,34]. As an immune stimulant, garlic components stimulate the proliferation of splenocytes [34,35] and synthesis of NO and TNF [36,37]. However, under certain circumstances, allicin or garlic extract may also work as an immune suppressant to down-regulate inflammatory responses and inhibit the interaction of T cells with the endothelial cells [38].

Although the anti-parasitic effects of garlic extract and allicin have been investigated, little is known about the immunomodulatory effects of garlic on parasitic infections. In *Leishmania major*-infected susceptible mice, treatment with garlic extract promoted the shift towards a Th1 response and enhanced the phagocytic activity of peritoneal macrophages, which significantly improved the disease outcome [39,40]. Here, the murine malaria model was used to investigate the effects of allicin on the course of infection of BALB/c mice with the lethal strain of *Plasmodium yoelii* 17XL. The results indicated that allicin treatments promoted the production of pro-inflammatory mediators and protected the host from *Plasmodium* infection.

## Methods

### Mice, parasite, and infection

Female, six to eight weeks old, BALB/c mice were purchased from Academia Sinica Shanghai experimental animal centre. *Plasmodium yoelii* 17XL infections were initiated by intraperitoneal (IP) injection of 1 × 10^6^*P. yoelii* 17XL parasitized red blood cells (pRBCs) per mouse. Parasitaemia was determined every other day by light microscopic examination of at least 1,000 erythrocytes on Giemsa-stained blood smears. Mortality was checked daily. All experiments were performed in compliance with local animal ethics committee requirements.

### Allicin treatment

Allicin was purchased from Jinkongfu Pharmaceutical (Wuhan, China). The stock solution was prepared by dissolving allicin in ethanol at a concentration of 10 mg/ml. It was diluted to 1 mg/ml with phosphate buffered saline (PBS) before use. For animal experiment, BALB/c mice were randomly divided into three groups. Allicin was orally administered by gavage at a dose of 3 or 9 mg/kg/day on days 0–2 post-infection (PI). Mice in the control group received 0.2 ml PBS at the same time points. Three mice in each group were sacrificed on day 3 and 5, respectively. The experiment was repeated three times.

### Spleen cell culture and quantification of cytokines

Spleens from BALB/c mice were removed aseptically and splenocytes were cultured as previously described [41]. Splenocytes were adjusted to a final concentration of 1 × 10^7^ cells/ml in RPMI1640 supplemented with 10% heat-inactivated foetal calf serum (FCS). Aliquots of the cell suspension (5 × 10^6^ cells/well) were seeded into 24-well, flat-bottom, tissue culture plates in triplicate, and incubated for 48 hr at 37°C in a humidified 5% CO_2_ incubator. The supernatants were collected and stored at −80°C until assayed for cytokines.

Levels of IFN-γ, TNF, IL-12p70, IL-4 and IL-10 were measured by commercial enzyme-linked immunosorbent assay (ELISA) kits according to the manufacturer's protocols (R&D Systems, Minneapolis, MN, USA). As a measure of NO production, concentrations of NO_2_^-^ in cell culture supernatants were determined by the Griess reaction [42].

### Flow cytometry

At the indicated time points, splenocytes were obtained from infected BALB/c mice for flow cytometric analysis to determine the subsets of spleen DCs: CD11c^+^CD11b^+^ myeloid DCs (mDCs) and CD11c^+^CD45R/B220^+^ plasmacytoid DCs (pDCs), the population of CD11c^+^DCs expressing MHCII or TLR9, macrophages and Treg. Antibodies and their sources were described previously [41]. Flow cytometry was performed on a FACS Calibur (BD Biosciences, San Diego, CA, USA) and analysed using the FlowJo software (Treestar, San Carlos, CA, USA).

### Statistical analysis

For each experiment, three mice were used to obtain an average, and the average values from three experiments were used to calculate the mean and standard error (SEM). Statistical analysis was performed by one-way ANOVA using the statistical software SPSS version 16.0. Fisher’s LSD post-hoc test was applied to assess differences of each group. Time-to-event data were statistically analysed with the Kaplan-Meier approach to survival analysis using the statistical software SPSS version 16.0. *P* values less than 0.05 were considered statistically significant.

## Results

### Allicin improves survival by reducing parasitaemia

**Figure 1:**
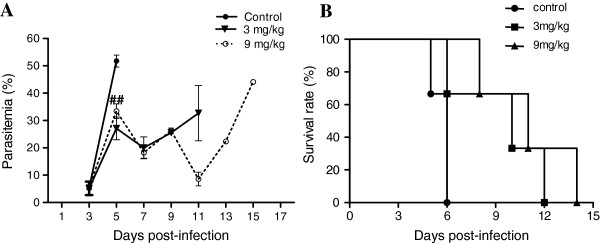
**Effects of allicin treatment on parasitaemia and survival of* P. yoelii* 17XL infected mice.** Mice were treated with two doses of allicin (3 and 9 mg/kg) and PBS (control group) for successive three days after * P. yoelii* 17 XL infection. Parasitaemia was calculated by counting the number of parasite-infected erythrocytes per 1,000 erythrocytes. Mortality was monitored daily. Results are presented as arithmetic mean of three mice per group ± the standard error of the mean (SEM). ##, significant difference (*P <* 0.001) compared to the control group.

The *P. yoelii* 17XL strain is highly virulent to BALB/c mice and causes lethal infection. In the control group, parasitaemia rose sharply and reached a peak level (51.8%) on day 5 PI (Figure 1A) and all mice died by day 7 (Figure 1B). In contrast, three-day oral allicin treatments at two dosages significantly reduced the day 5 parasitaemias (27.1% and 32.6% for the 3 and 9 mg/kg groups, respectively). Further decline of the parasitaemia was noticed on day 7 PI in both allicin treatment groups (Figure 1A). Consistent with an earlier observation on the effect of allicin on *Plasmodium berghei* erythrocytic infection [30], allicin treatments at both dosages significantly extended the survival time of infected mice (*P <* 0.05 and *P <* 0.01 compared to NC group by Kaplan-Meier’s statistics, respectively); all mice died by day 13 and 15, respectively (Figure 1B). 

### Allicin preferentially enhances pro-inflammatory immune responses

**Figure 2:**
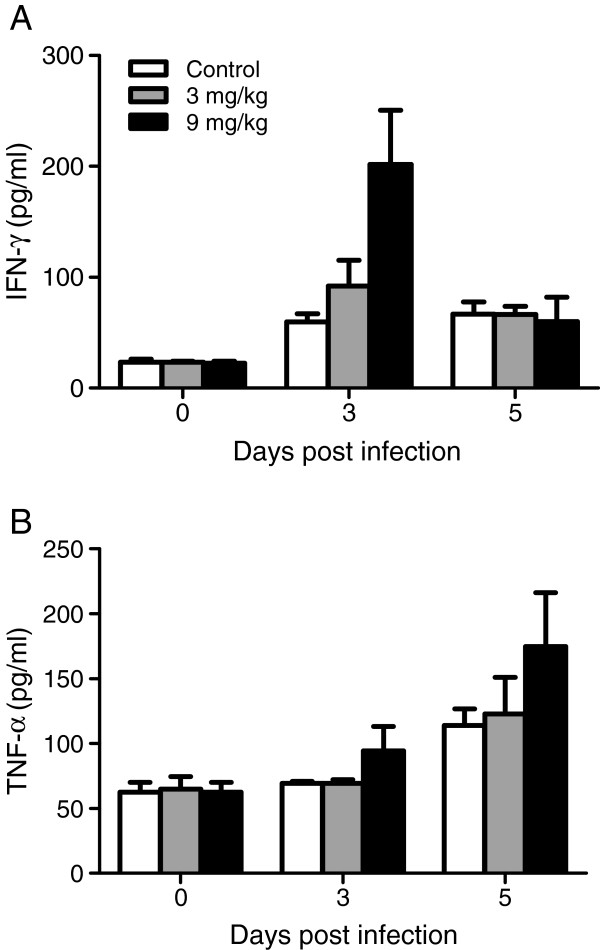
**Serum levels of pro-inflammatory cytokines IFN-γ and TNF.** Sera were collected from allicin and PBS treated mice and the amounts of IFN-γ (**A**) and TNF (**B**) were assayed by ELISA on day 0, 3 and 5 PI. There was no significant difference among groups by Fisher’s LSD post-hoc test.

**Figure 3:**
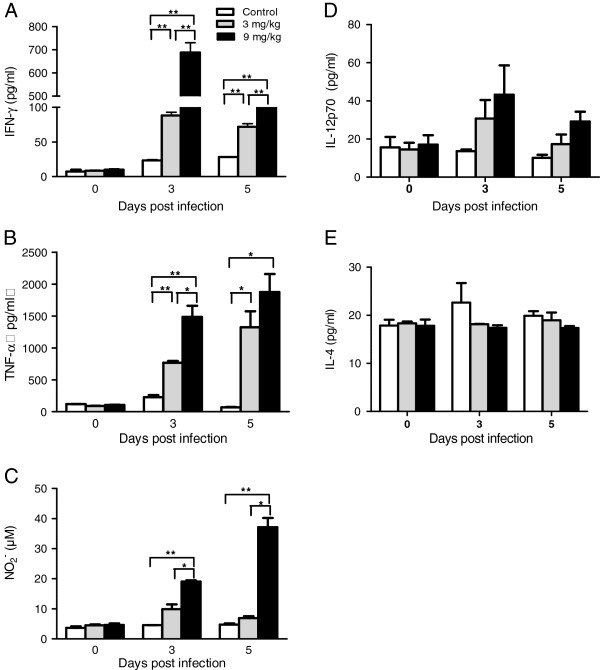
**Effects of allicin on pro-inflammatory immune responses during murine malaria infection.** On day 0, 3 and 5 after infection, spleen cells were prepared and concentrations of IFN-γ (**A**), TNF (**B**), IL-12p70 (**D**) and IL-4 (**E**) were determined by ELISA. The concentration of NO_2_^-^ (**C**) was detected using the Griess reaction.

As a cysteine protease inhibitor, the inhibitory effects of allicin on *Plasmodium* parasites were attributed to the direct action on parasites [30,32]. Because allicin also has immunomodulatory activity, whether improved disease outcomes by allicin treatments could result from strengthened host immunity against *Plasmodium* infection was investigated. Previous studies have shown that enhancement of Th1 responses during *P. yoelii* 17XL infection could reduce the initial parasite load and extend host survival time [43]. Here, the levels of several pro-inflammatory mediators in the sera of control and allicin-treated mice were evaluated. Allicin treatments increased IFN-γ levels on day 3 PI and treatment at 9 mg/kg increased TNF levels on both days 3 and 5 PI, although the differences were not statistically significant (Figure 2). To further investigate whether the elevated serum levels of pro-inflammatory cytokines were the result of increased production in splenocytes, the *in vitro* synthesis of IFN-γ, TNF, IL-12p70 and NO in cultured splenocytes from the control and allicin-treated mice were measured. Compared to the control, allicin treatments at both dosages caused significant increases in the production of IFN-γ and TNF by splenocytes on days 3 and 5 PI (*P* < 0.05) and the effect appeared to be dose-dependent (Figure 3A, B). More specifically, 9 mg/kg allicin treatment led to ~ seven times higher production of IFN-γ than 3 mg/kg allicin treatment on day 3 PI (Figure 3A). IFN-γ can promote the production of NO by macrophages to reduce the parasitaemia during *P. yoelii* 17XL infection. Therefore, NO production in cultured splenocytes, a hallmark of macrophage activation, was further studied. In support of earlier observation of other Th1 cytokines, both allicin treatment dosages increased NO production on days 3 and 5 PI (Figure 3C). Yet, the 3 mg/kg treatment group showed slight, insignificant increase of NO production as compared to control (Figure 3C, *P* > 0.05). Significant increase in NO_2_^-^ was evident in the higher dose (9 mg/kg) of allicin treatment group (Figure 3C). IL-12 is an important stimulator of the T-cell response and plays a critical role in resistance to malaria [44-46]. Allicin treatments at both dosages caused increases in the production of IL-12p70 by splenocytes on days 3 and 5 PI and this effect also appeared to be dose-dependent, albeit the difference was not statistically significant (Figure 3D, *P* > 0.05). To evaluate whether allicin treatment affected Th2 immune response during the early stage of *P. yoelii* 17XL infection, the amount of IL-4 in the supernatant of cultured splenocytes was determined, and there was no significant difference between the experiment and control groups (Figure 3E, *P* > 0.05). Altogether, these results showed that allicin treatment preferentially promoted the production of pro-inflammatory mediators during acute malaria infection in a dose-dependent manner. 

### Allicin treatments stimulate expansion of CD4^+^ T cells and macrophages

**Figure 4:**
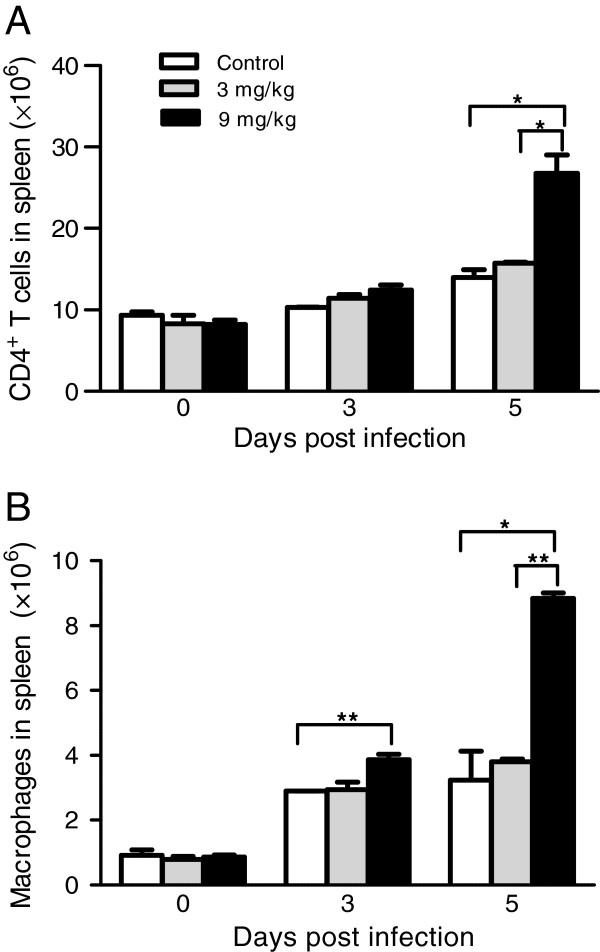
**Effects of allicin treatments on CD4**^**+**^**T cells and macrophages during* P. yoelii* 17XL infection.** Absolute numbers of CD4^+^ T cells (**A**) and macrophages (**B**) were quantified by flow cytometry at day 0, 3 and 5 PI. One experiment representative of three is shown. Error bars represents SEM. Asterisks indicate statistically significant differences (*: *P <* 0.05; **: *P <* 0.01) between groups.

Protective immunity against blood-stage *Plasmodium* requires malaria-specific CD4^+^ T cells to rapidly and effectively control parasitaemia and clear the infection [47]. In addition, macrophages also play an essential role for parasite control during the early acute phase infection by the lethal *P. yoelii* strain [9]. Whereas significant changes in the number of CD4^+^ T cells in both allicin treatment groups on day 3 PI were observed, spleen CD4^+^ T cell expansion was detected on day 5 PI (Figure 4A). Again, this change in spleen CD4^+^ T cell was only evident in the 9 mg/kg allicin-treated group. Similarly, mice treated with 9 mg/kg of allicin had significantly more macrophages in the spleen than either control or 3 mg/kg allicin-treated mice (Figure 4B). 

### Allicin treatments promote the activation of dendritic cells

**Figure 5:**
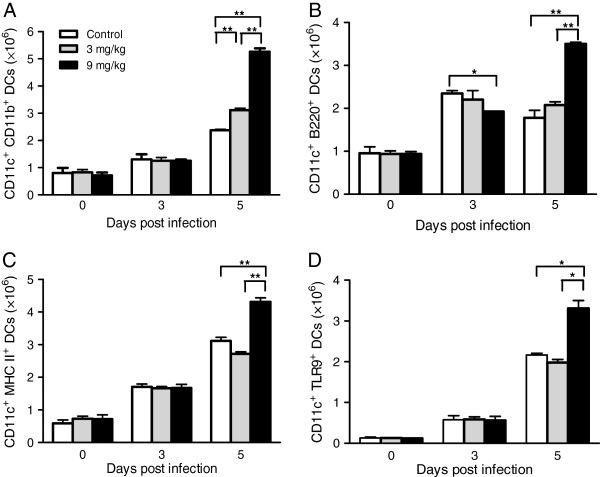
**Effects of allicin treatments on DCs during* P. yoelii* 17XL infection.** Representative graphs show the absolute numbers of cells in the spleens for (**A**) mDCs, (**B**) pDCs, and (**C**) CD11c^+^ MHCII^+^ DCs, (**D**) CD11c^+^ TLR9^+^ DCs on day 0, 3, and 5 PI. The cells were quantified by flow cytometry analysis and data presented as the mean ± standard error (n = 3 mice/group). Results are representative of three independent experiments. Error bars represents SEM. Asterisks indicate statistically significant differences (*: *P <* 0.05; **: *P <*0.01) between groups.

DCs are the critical link between innate and adaptive immune responses. Two subpopulations of DCs are defined as mDCs (CD11c^+^CD11b^+^) and pDCs (CD11c^+^CD45R/B220^+^). On day 3 PI, the numbers of mDCs were not significantly different between the allicin treatment groups and control (Figure 5A). However, 9 mg/kg allicin significantly suppressed the total number of spleen pDCs (Figure 5B). On day 5 PI, allicin treatment at 3 mg/kg produced more mDCs and pDCs, albeit the increases were not statistically significant. In comparison, the numbers of both DC populations on day 5 PI in the 9 mg/kg allicin treatment group were significantly higher than those in the control or 3 mg/kg allicin treatment groups (Figure 5A, B).

Maturation of DCs is essential to induce Th1 immune response for antigen presentation to T cells [48]. To study whether allicin treatment had any effect on activation of DCs, the numbers of DCs expressing MHCII and TLR9 between the control and treatment groups were compared. Infection with *P. yoelii* 17XL significantly increased the total numbers of mature DCs expressing MHC II on day 3 PI, but no significant difference was observed among the treatment groups (Figure 5C). On day 5 PI, however, 9 mg/kg allicin treatment group produced significantly more MHC II-expressing DCs than control (*P <* 0.05) or 3 mg/kg allicin treatment group (*P <* 0.01) (Figure 5C). Analysis of TLR9-expressing DCs revealed a similar trend as that of the MHCII-expressing DCs (Figure 5D). Collectively, these results indicate that allicin treatment at 9 mg/kg promoted expansion of matured DCs and enhanced TLR9-mediated innate immune activation on day 5 during *P. yoelii* 17XL infection.

### Allicin has no effect on IL-10 production or Treg

**Figure 6:**
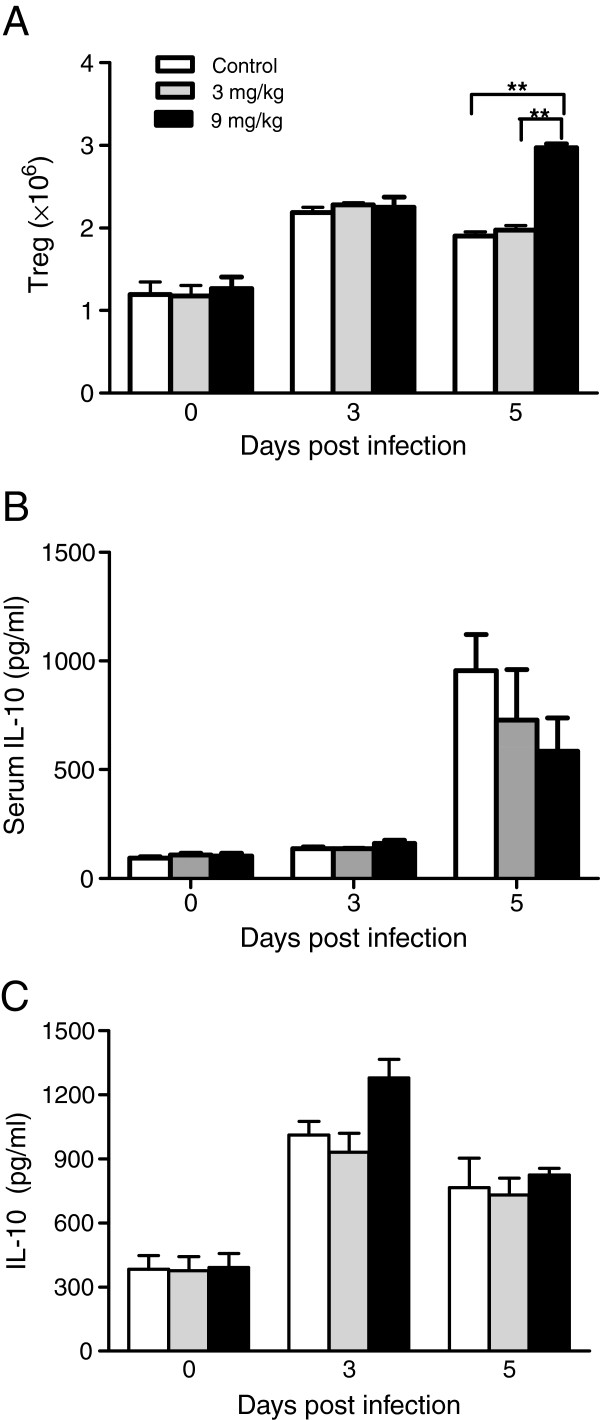
**Effects of allicin treatments on Treg and IL-10 level during* P. yoelii* 17XL infection.** (**A**) Absolute numbers of Treg at various time points as detected by flow cytometry analysis. (**B**) The concentrations of IL-10 in sera on day 3 and 5 PI. (**C**) The concentrations of IL-10 produced by cultured splenocytes. Concentrations of IL-10 were determined by ELISA. Data expressed as the mean with SEM (n = 3 mice per group). Results are representative of three independent experiments. Asterisks indicate statistically significant differences (**: *P <* 0.01) between groups.

The immunomodulatory functions of allicin can also down-regulate the inflammatory response [49-51]. The effect of allicin treatment on Treg, which has an inhibitory effect on Th1 immune response, and the anti-inflammatory cytokine IL-10 was examined. Only allicin treatment at 9 mg/kg significantly increased the absolute numbers of Treg on day 5 PI as compared to both control mice and 3 mg/kg allicin-treated mice (Figure 6A). However, the serum levels of IL-10 were not significantly different between the control and allicin-treated group on either day 3 or day 5 PI (Figure 6B). Similarly, the production of IL-10 by cultured splenocytes was not significantly different between the control and allicin-treated groups (Figure 6C). 

## Discussion

Allicin, a sulphur compound produced in garlic, has antibacterial, antifungal and antiparasitic activities. The main mechanism and mode of action of allicin is generally considered to be its reaction with the SH group on cysteine residues of enzymes in the pathogens, resulting in their inactivation [52,53]. The antiparasitic activity of allicin on *Plasmodium* and *Trypanosoma* was attributed to the inhibition of cysteine proteases in these parasites [32]. Previous studies have shown that allicin inhibited *P. berghei* circumsporozoite protein processing and prevented sporozoite invasion of host cells *in vitro* as well as protected the *P. berghei*-infected mice from early death [30,54]. This study assessed the immunomodulatory effect of allicin on *P. yoelii* 17XL-infected mice. The results showed the anti-malarial activity of allicin in *P. yoelii* 17XL infected mice was partially due to its enhancement of the pro-inflammatory immune response by expanding the populations of CD4^+^ T cells, mDCs and macrophages as well as stimulating DCs maturation.

Pro-inflammatory mediators play an important role in controlling parasitaemia at the early stage during *P. yoelii* 17XL infection [6,11,55,56]. Expansion of macrophages and elevated TNF level are critical for controlling parasitaemia [57], while IFN-γ forms a central mediator of protective immune responses against pre-erythrocytic and blood-stage malaria parasites [58]. During the acute phase of malaria infection, native T cells may be stimulated to produce IFN-γ and TNF [59]. Allicin treatment significantly elevated the levels of these pro-inflammatory mediators (IFN-γ, TNF and NO) in a dose-dependent manner in *P. yoelii* 17XL-infected BALB/c mice, consistent with allicin’s function as an immune stimulant [34-37]. To a lower extent, allicin treatment also enhanced the production of IL-12p70 of cultured spleen cells, another indicator for enhanced Th1 response. Finally, allicin treatment stimulated expansion of CD4^+^ T cells, which further supports the activation of Th1 immune response. As a hallmark of Th2 immune response, IL-4 level in allicin treated mice was comparable to that in control mice, indicating that allicin treatment did not affect Th2 immune response during early *P. yoelii* 17XL infection.

DCs bridge the innate and adaptive immune response as APCs via antigen presentation to helper T cells, which can activate native T cells and polarize CD4^+^ T cells response [60]. Stimulation of T-cell responses, and more importantly, induction of Th1 cell development, is associated with maturation of DCs as well as their production of Th1 cytokines [61,62]. Thus, the strategy to improve the maturation and activation of DCs is key to the initiation of a protective immune response against malaria infection. The results suggested that allicin treatment could significantly promote the maturation of DCs with increasing expression of the co-stimulatory molecules.

Toll like receptors (TLRs) expressed on the innate immune cells (such as DCs) engaged in the recognition of constituents of protozoan parasites [63,64]. Upon TLR-driven activation, DCs produce pro-inflammatory and protective cytokines that contribute to innate immunity. TLR9 mediates innate immune activation by the malaria haemozoin [65] and protein-DNA complex [66]. TLR9 mediates parasite recognition and initiates IFN-γ production to prime host innate responses against malaria [67,68]. In summary, allicin could expand the population of TLR9-expressing DCs, resulting in increases of the IFN-γ level.

Another aspect of allicin’s immunomodulatory effect is down-regulation of pro-inflammatory response. Allicin could reduce the TNF level in a dose-dependent manner and suppress both spontaneous and TNF stimulated secretion of cytokines IL-1, IL-6 and IL-8 [38,69]. This is largely due to regulation of the host Treg and anti-inflammatory cytokine IL-10. Depletion of Treg protects BALB/c mice infected with *P. yoelii* 17XL from overwhelming parasitaemia and death [70]. Therefore, Treg provide an essential mechanism for the parasites to evade host-mediated immunity. In addition, TLR9 engagement in DCs is required for natural Treg activation by malaria parasites [71]. The higher dose of allicin treatment increased the TLR9 expression on DCs on day 5 PI, which in turn increased the number of Treg. However, the level of IL-10 was not correspondently elevated, suggesting that allicin treatment did not drastically modify the function of Treg during the acute malaria infection.

In summary, allicin treatment can protect host against malaria infection by activating pro-inflammatory immune responses in a dose-dependent manner. The immune-stimulatory effect of allicin is characterized by induced mature DCs during early phase of *P. yoelii* 17XL infection, which leads to increased levels of pro-inflammatory mediators from proliferative macrophages and CD4^+^ T cells. The results provide important insights into the *in vivo* parasite-inhibitory mechanism of allicin, which suggests the involvement of both direct inhibition of parasite enzymes and stimulation of antiparasitic immune response of the host.

## Conclusions

Allicin treatment could enhance host immunity against malaria infection in a rodent malaria model. It was observed that treatment with allicin during *P. yoelii* 17XL infection could enhance host innate and adaptive immunity evidenced by elevated numbers of macrophages and CD4^+^ T cells and cytokines. In addition, allicin treatment promoted the expansion and maturation of DCs, which play an essential role in initiating adaptive immunity. However, the function of Treg was not altered by allicin treatment. Collective findings from this study suggest that allicin partially protects host against malaria infection through enhancement of the host’s innate and adaptive immune responses.

## Competing interests

The authors declare that they have no competing interests.

## Authors’ contributions

YF carried out the flow cytometry, statistical analysis and drafted the manuscript. XZ performed detection of cytokines and NO_2_^-^ concentration. YJ and HS helped to revise the manuscript. YC and LC conceived the study and participated in the design of the study. All authors read and approved the final manuscript.
